# Intraocular Pressure and Estimated Cerebrospinal Fluid Pressure. The Beijing Eye Study 2011

**DOI:** 10.1371/journal.pone.0104267

**Published:** 2014-08-08

**Authors:** Ya Xing Wang, Jost B. Jonas, Ningli Wang, Qi Sheng You, Diya Yang, Xiao Bin Xie, Liang Xu

**Affiliations:** 1 Beijing Institute of Ophthalmology, Beijing Tongren Eye Center, Beijing Tongren Hospital, Capital Medical University, Beijing Ophthalmology and Visual Science Key Lab, Beijing, China; 2 Department of Ophthalmology, Medical Faculty Mannheim of the Ruprecht-Karls-University of Heidelberg, Heidelberg, Germany; 3 Beijing Tongren Eye Center, Beijing Tongren Hospital, Capital Medical University, Beijing Ophthalmology and Visual Sciences Key Laboratory, Beijing, China; Centre for Eye Research Australia, Australia

## Abstract

**Purpose:**

To examine a potential association between intraocular pressure (IOP) and cerebrospinal fluid pressure (CSFP) in a population-based setting.

**Methods:**

The population-based Beijing Eye Study 2011 included 3468 individuals with a mean age of 64.6±9.8 years (range: 50–93 years). A detailed ophthalmic examination was performed. Based on a previous study with lumbar cerebrospinal fluid pressure (CSFP) measurements, CSFP was calculated as CSFP [mm Hg] = 0.44×Body Mass Index [kg/m^2^]+0.16×Diastolic Blood Pressure [mm Hg]–0.18×Age [Years].

**Results:**

In multivariate analysis, IOP was associated with higher estimated CSFP (P<0.001; standardized correlation coefficient beta: 0.27; regression coefficient B: 0.20; 95% confidence interval (CI): 0.16, 0.24), after adjusting for thinner central corneal thickness (P<0.001; beta: 0.45; B: 0.04;95%CI: 0.04,0.04), smaller corneal curvature radius (P<0.001; beta:−0.11; B:−1.13;95%CI:−1.61,−0.64), shallower anterior chamber depth (P = 0.01; beta:−0.05; B:−0.33;95%CI:−0.59,−0.08) and longer axial length (P = 0.002; beta: 0.08; B: 0.20;95%CI: 0.08,0.32)), and after adjusting for the systemic parameters of higher pulse rate (P<0.001; beta: 0.08; B: 0.02;95%CI: 0.01,0.03), higher prevalence of arterial hypertension (P = 0.002; beta: 0.06; B: 0.32;95%CI: 0.12,0.53)), frequency of drinking alcohol (P = 0.02; beta: 0.04; B: 0.09;95%CI: 0.01,0.17), higher blood concentration of triglycerides (P = 0.001; beta: 0.06; B: 0.06;95%CI: 0.02,0.10) and cholesterol (P = 0.049; beta: 0.04; B: 0.08;95%CI: 0.00,0.17), and body mass index (P<0.001; beta:−0.13; B:−0.09;95%CI:−0.13,−0.06). In a parallel manner, estimated CSFP (mean: 10.8±3.7 mm Hg) was significantly associated with higher IOP (P<0.001; beta: 0.13; B: 0.18;95%CI: 0.13,0.23) after adjusting for rural region of habitation (P<0.001; beta:−0.37; B:−2.78;95%CI:−3.07,−2.48), higher systolic blood pressure (P<0.001; beta: 0.34; B: 0.06;95%CI: 0.05,0.07), higher pulse rate (P = 0.003; beta: 0.05; B: 0.02;95%CI: 0.01,0.03), taller body height (P<0.001; beta: 0.11; B: 0.05;95%CI: 0.03,0.07), higher blood concentration of cholesterol (P = 0.003; beta: 0.05; B: 0.17;95%CI: 0.06,0.28) and higher level of education (P = 0.003; beta: 0.09; B: 0.30;95%CI: 0.16,0.45).

**Conclusions:**

IOP was positively associated with estimated CSFP after adjusting for other ocular and systemic parameters. As a corollary, higher estimated CSFP was significantly associated with higher IOP in multivariate analysis. It fits with the notion that the arterial blood pressure, estimated CSFP and IOP are physiologically correlated with each other.

## Introduction

Previous studies have suggested a physiologic correlation between cerebrospinal fluid pressure (CSFP), blood pressure and intraocular pressure (IOP) [1]–[3]. These studies included patients who underwent a lumbar puncture with direct measurement of the lumbar CSFP for neurological reasons and for whom the final neurological diagnosis made it likely that the neurological or neuro-ophthalmological diseases had not influenced the CSFP. Population-based studies revealed that IOP was correlated with blood pressure [4]. Other investigations showed an association between CSFP and body mass index which was associated with higher arterial blood pressure [5], [6]. Combining the findings of the various studies led to the hypothesis, that the pressures in all three fluid-filled compartments, i.e. the arterial blood system, the cerebrospinal fluid compartment and the intraocular space, were correlated with each other. The studies had however limitations, such as that the investigations with direct lumbar puncture did not include normal subjects and that the number of study participants was relatively small [1]–[3]. To test the hypothesis that IOP and CSFP are correlated with each other, we performed a population-based study to assess an association between both pressure parameters. We chose a population-based study design to avoid a potential bias due to referral-related selection of study participants. We estimated the CSFP based on diastolic blood pressure, age and body mass index, using a formula which was derived in a previous pilot investigation on the relationship between these three parameters [3]. Since IOP is related to a magnitude of ocular and systemic parameters, we assessed the potential association between IOP and estimated CSFP in a multivariable analysis [4]–[12].

## Methods

The Beijing Eye Study 2011 is a population-based cross-sectional study in Northern China [13], [14]. The Medical Ethics Committee of the Beijing Tongren Hospital approved the study protocol and all participants gave informed consent. The only eligibility criterion for inclusion into the study was an age of 50+ years. Out of an eligible population of 4403 individuals, 3468 (78.8%) individuals (1963 (56.6%) women) participated. The study has been described in detail previously [13], [14]. Intraocular pressure was measured using a non-contact pneumotonometer (CT-60 computerized tonometer, Topcon Ltd., Japan) by an experienced technician. Three measurements were taken, and the mean of the three measurements was taken for further statistical analysis. If the measurements were higher than 25 mm Hg, tonometry was repeated.

Using the lumbar CSF-P measurements obtained in a previous pilot study on neurological patients, we assessed the associations between lumbar CSFP measurements, diastolic blood pressure, body mass index and age [3]. The indications for lumbar puncture in that pilot study were peripheral neuropathy, intracranial hypertension, spontaneous intracranial hypotension, cavernous sinus syndrome, meningitis, multiple sclerosis, unilateral ischemic optic neuropathy, unilateral optic neuritis, optic nerve atrophy, and head injury. The study included 74 patients with a mean age of 42.0±13.4 years. All measured CSFP values were less than 24.3 mm Hg. Out of the total group, we randomly formed a training group consisting of 32 patients, and a group including the remaining 42 patients. Performing a multivariate analysis in the training group with the lumbar CSFP measurements as dependent variable and age, body mass index and blood pressure as independent variables revealed, that CSFP was best described by the formula as CSFP [mm Hg] = 0.44×Body Mass Index [kg/m^2^]+0.16×Diastolic Blood Pressure [mm Hg]–0.18×Age [Years]−1.91. We then tested the formula in the test group. In this test group which was independent of the training group, the measured lumbar CSFP (12.6±4.8 mm Hg) did not differ significantly (*P* = 0.29) from the calculated CSFP (13.3±3.2 mm Hg). The Durbin-Watson value was 2.08. Values falling into the acceptable range of 1.5 to 2.5 indicate a non-significant autocorrelation for the residuals in the multiple regression models. The intra-class correlation coefficient was 0.71. The Bland-Altman analysis revealed that 40 out of 42 measurements were within the 95% limits of agreement. If the test group was taken as training group, the algorithm to calculate the CSFP was CSFP [mm Hg] = 0.85×Body Mass Index [kg/m^2^]+0.27×Diastolic Blood Pressure [mm Hg]–0.08×Age [Years]−24.8.

Inclusion criterion for the present study was the availability of IOP measurements, body mass index values and diastolic blood pressure measurements. Statistical analysis was performed using a commercially available statistical software package (SPSS for Windows, version 21.0, IBM-SPSS, Chicago, IL). First, we examined the mean values (presented as mean ± standard deviation). Second, we searched for associations between IOP, estimated CSFP and other systemic and ocular parameters in univariate analysis. Third, we performed a multivariate analysis, with IOP as dependent variable and all those parameters as independent variables, which had a significant association with IOP in the univariate analysis. We then dropped step-by-step those parameters which were no longer significantly associated with IOP, starting with the parameters with the highest *P*-values. Fourth, in a reverse manner, we checked for associations between CSFP and other systemic and ocular parameters including IOP. All *P*-values were 2-sided and were considered statistically significant when the values were less than 0.05; 95% confidence intervals (CI) were presented.

## Results

Measurements of IOP, blood pressure and body mass index were available for 6684 eyes of 3353 (96.7%) subjects with a mean age of 64.4±9.7 years (median: 63 years; range: 50 to 93 years), a mean refractive error of −0.22±2.10 diopters (median: 0.25 diopters; range: −22.0 to +7.00 diopters), and a mean axial length of 22.3±1.1 mm (median: 23.1 mm; range: 18.96–30.88 mm). The group of subjects not participating in the study as compared with the group of subjects included into the study was significantly (*P*<0.001) older (69.8±11.4 years versus 64.4±9.7 years), while both groups did not differ significantly in refractive error (−0.31±3.96 diopters versus −0.22±2.10 diopters; *P* = 0.93), axial length (23.1±1.8 mm versus 22.3±1.1 mm; *P* = 0.75) nor gender (*P* = 0.06). Glaucomatous optic neuropathy was detected in 385 (5.4%) eyes, with 256 (3.8%) eyes with open-angle glaucoma, 125 (1.9%) eyes with primary angle-closure glaucoma, and 4 (0.1%) eyes with secondary angle-closure glaucoma.

**Figure 1:**
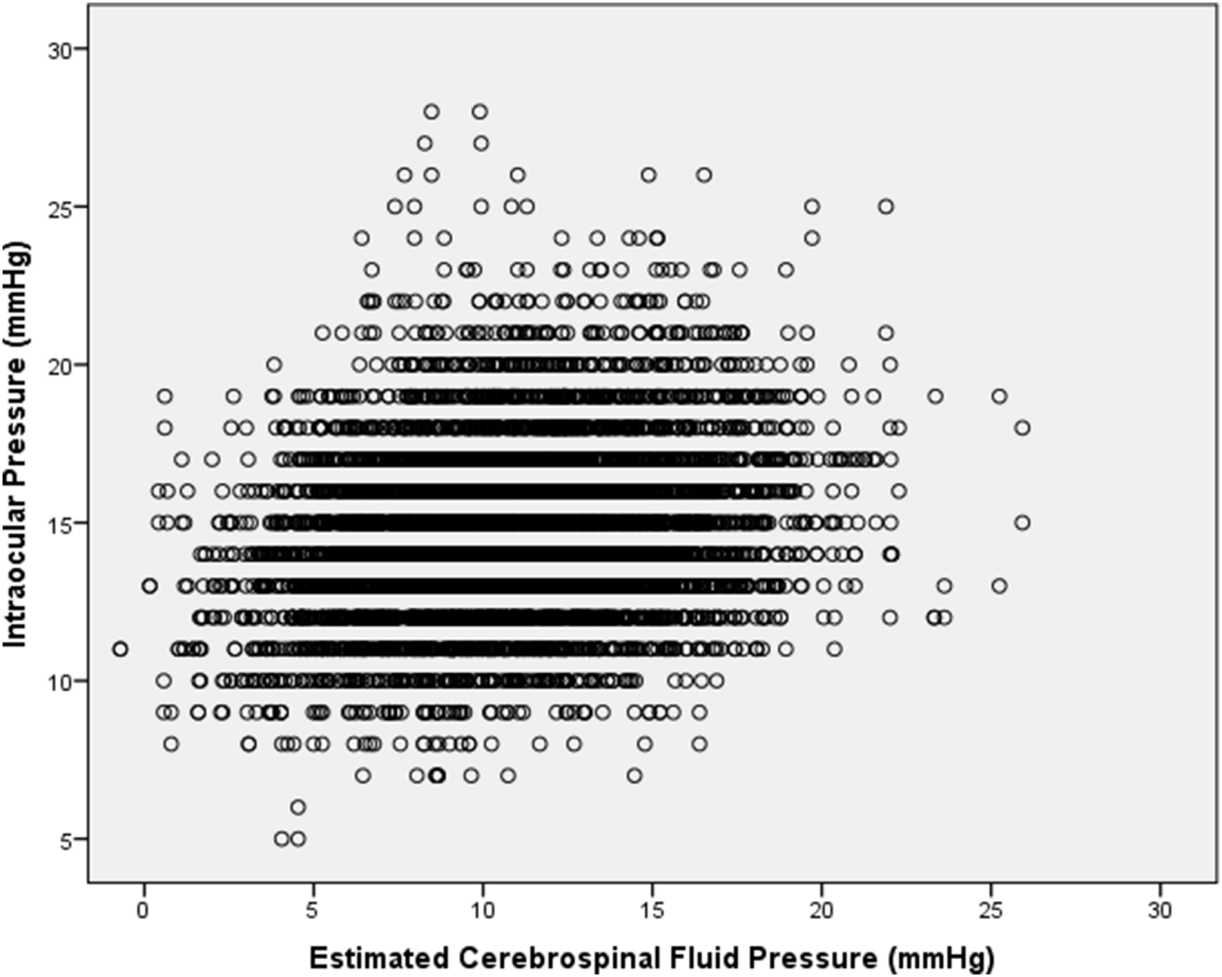
Scattergam showing the distribution of intraocular pressure and estimated cerebrospinal fluid pressure in the non-glaucomatous population of the Beijing Eye Study 2011.

After excluding subjects with glaucoma, mean IOP was 14.7±2.8 mm Hg. In univariate analysis, higher IOP was significantly (*P*<0.05) associated with the numerous ocular parameters and systemic parameters including higher estimated CSFP (Fig. 1) (Table 1).

**Table 1:** Associations (univariate analysis) between the intraocular pressure and ocular and systemic parameters in the Beijing Eye Study 2011.

Parameter	*P*-Value	Standardized CorrelationCoefficient Beta	Regression Coefficient B orMean Difference	95% Confidence Interval
**Systemic Parameters**				
Age	<0.001	−0.16	−0.05	−0.05, −0.04
Gender	0.07			
Rural/Urban Regionof Habitation	<0.001	−0.09	−0.48	−0.62, −0.35
Level if Education	0.72			
Body Height (cm)	0.06			
Body Weight (kg)	<0.001	0.09	0.02	0.01, 0.03
Body Mass Index (kg/m^2^)	<0.001	0.08	0.05	0.03, 0.08
Waist Circumference (cm)	<0.001	0.08	0.02	0.01, 0.03
Blood ConcentrationGlucose (mmol/L)	<0.001	0.09	0.16	0.11, 0.21
Glycosylated HemoglobinHbA1c (%)	<0.001	0.08	0.22	0.15, 0.30
Diabetes Mellitus	<0.001	0.06	0.43	0.24, 0.63
Blood ConcentrationHigh-Density Lipoprotein	0.91			
Blood ConcentrationLow-Density Lipoproteins	0.04	0.04	0.12	0.00, 0.24
Blood ConcentrationTriglycerides	<0.001	0.09	0.09	0.05, 0.14
Blood Concentration Cholesterol	<0.001	0.07	0.17	0.10, 0.24
Systolic BloodPressure (mm Hg)	<0.001	0.13	0.02	0.01, 0.02
Diastolic BloodPressure (mm Hg)	<0.001	0.19	0.04	0.04, 0.05
Mean BloodPressure (mm Hg)	<0.001	0.18	0.04	0.03, 0.04
Arterial Hypertension	<0.001	0.07	0.38	0.25, 0.52
Pulse Rate (Beats/min)	<0.001	0.08	0.02	0.02, 0.03
Estimated CerebrospinalFluid Pressure (mm Hg)	<0.001	0.22	0.16	0.14, 0.18
Smoking PackageYears	<0.001	0.08	0.11	0.06, 0.16
Smoking (Ever)	<0.001		0.40	0.19, 0.61
Alcohol ConsumptionFrequency	<0.001	0.07	0.10	0.05, 0.16
Aspirin Intake	0.17			
Ocular Parameters				
Refractive Error (Diopters)	0.006	−0.05	−0.03	−0.08, −0.01
Corneal Thickness (µm)	<0.001	0.44	0.04	0.03, 0.04
Anterior Corneal Curvature (mm)	0.23			
Anterior ChamberDepth (mm)	0.001	−0.06	−0.33	−0.54, −0.13
Lens Thickness (mm)	0.01	−0.05	−0.38	−0.67, −0.09
Axial Length (mm)	0.37			
Optic Disc Area (mm^2^)	0.81			
Neuroretinal Rim Area	0.04	−0.05	−0.34	−0.67, 0.01
Parapapillary Atrophy, Alpha Zone (mm^2^)	0.25			
Parapapillary Atrophy, Beta Zone (mm^2^)	0.86			
Early Age-RelatedMacular Degeneration	0.02	−0.03	−0.30	−0.54, −0.06
Diabetic Retinopathy	0.02	0.03	0.52	0.09, 0.95
Retinal Vein Occlusion	0.64			

In the multivariate analysis, we included as independent variables all parameters, which were significantly associated with IOP in the univariate analysis. We first dropped parameters with a high collinearity (i.e., a variance inflation factor >2.5: body weight, waist circumference, systolic blood pressure, ever smoking). We then dropped step by step parameters which were no longer significantly associated with IOP. IOP was eventually associated with higher estimated CSFP (*P*<0.001; standardized correlation coefficient beta: 0.10; regression coefficient B: 0.07 (95%CI: 0.03, 0.11)), after adjusting for the ocular parameters of thinner central corneal thickness (*P*<0.001; beta: 0.46; B: 0.04 (95%CI: 0.04,0.04)), shorter corneal curvature radius (*P<*0.001; beta:−0.10; B:−1.12 (95%CI:−1.60,−0.64)), shallower anterior chamber depth (*P* = 0.008; beta:−0.06; B:−0.34 (95%CI:−0.60,−0.09)) and longer axial length (*P* = 0.001; beta: 0.08; B: 0.20 (95%CI: 0.08,0.33)), and after adjusting for the systemic parameters of younger age (*P*<0.001; beta:−0.12; B:−0.04 (95%CI:−0.05,−0.02)), higher pulse rate (*P*<0.001; beta: 0.09; B: 0.02 (95%CI: 0.01,0.03)), higher prevalence of arterial hypertension (*P<*0.001; beta: 0.09; B: 0.51 (95%CI: 0.28,0.74)), higher blood concentration of triglycerides (*P* = 0.002; beta: 0.06; B: 0.06 (95%CI: 0.02,0.10)) and cholesterol (*P* = 0.0.04; beta: 0.04; B: 0.09 (95%CI: 0.01,0.17)), and higher frequency of drinking alcohol (*P* = 0.007; beta: 0.05; B: 0.10 (95%CI: 0.03,0.18)). In this model of multivariate analysis, the highest overall correlation coefficient of r = 0.53 was achieved.

If age was dropped and body mass index was added to the list of independent variables, the weight of estimated CSFP in the model increased (i.e., the standardized correlation coefficient increased from 0.10 to 0.27), while the overall correlation coefficient remained unchanged (r = 0.53). If additionally body mass index and arterial hypertension were dropped (since they were included in the CSFP formula), higher IOP remained to be significantly correlated with higher estimated CSFP (*P*<0.001; beta: 0.20; B: 0.15 (95%CI: 0.12,0.18)).

Interestingly, the association between IOP and pulse rate in the multivariate analysis was considerably stronger in the group of subjects with arterial hypertension (*P*<0.001; beta: 0.10; B: 0.03;95%CI: 0.01,0.04) than in the group of subjects without arterial hypertension (*P* = 0.04; beta: 0.06; B: 0.02;95%CI: 0.00,0.03). The association between IOP and estimated CSFP was mostly unchanged (*P*<0.001; beta: 0.27; B: 0.18;95%CI: 0.13,0.24) versus (*P*<0.001; beta: 0.26; B: 0.23;95%CI: 0.16,0.29). In the whole study group, pulse rate itself was weakly associated with diastolic blood pressure (*P*<0.001; r: 0.08), but not with systolic blood pressure (*P* = 0.10) or with arterial hypertension (*P* = 0.09).

In the non-glaucomatous study population, mean estimated CSFP was 10.8±3.7 mm Hg and showed a Gaussian distribution curve (Kolmogorov-Smirnov-test; *P* = 0.74). As also shown in a previous investigation (own data), estimated CSFP was significantly associated (univariate analysis) with younger age (*P*<0.001), rural region (*P*<0.001), taller body height (*P*<0.001), higher body weight (*P*<0.001), higher body mass index (*P*<0.001), longer waist circumference (*P*<0.001), higher diastolic blood pressure (*P*<0.001) and systolic blood pressure (*P*<0.001), higher pulse (*P*<0.001), lower level of education (*P*<0.001), and higher prevalence of arterial hypertension (*P*<0.001), and higher blood concentration of glucose (*P* = 0.002), glycosylated hemoglobin (*P* = 0.002), trigylcerides (*P*<0.001), low-density lipoproteins (*P*<0.001) and cholesterol (*P*<0.001), and with the ocular parameters of shorter axial length (*P*<0.001), thinner central cornea (*P* = 0.02), refractive error (*P* = 0.02), higher IOP (*P*<0.001) and thicker lens (P<0.001).

The multivariate analysis included estimated CSFP as dependent variable and all those parameters which were significantly associated with estimated CSFP in the univariate analysis, except of age, blood pressure and body mass index. The three latter parameters had been used to calculate the CSFP. We then dropped out of the list of independent parameters those variables, which were no longer significantly associated with estimated CSFP, starting with the parameters with the highest *P*-values. Higher estimated CSFP was eventually associated with the systemic parameters of rural region of habitation (*P*<0.001; beta:−0.37; B:−2.78;95%CI:−3.07,−2.48), higher systolic blood pressure (*P*<0.001; beta: 0.34; B: 0.06;95%CI: 0.05,0.07), higher pulse rate (*P* = 0.003; beta: 0.05; B: 0.02;95%CI: 0.01,0.03), taller body height (*P*<0.001; beta: 0.11; B: 0.05;95%CI: 0.03,0.07), higher blood concentration of cholesterol (*P* = 0.003; beta: 0.05; B: 0.17;95%CI: 0.06,0.28), and higher level of education (*P* = 0.003; beta: 0.09; B: 0.30;95%CI: 0.16,0.45), and with the ocular parameter of higher IOP (*P*<0.001; beta: 0.13; B: 0.18;95%CI: 0.13,0.23).

## Discussion

In our population-based study, IOP was significantly (*P*<0.001) associated with higher estimated CSFP after adjusting for the ocular parameters of thinner central corneal thickness, shorter corneal curvature radius, shallower anterior chamber depth and longer axial length, and after adjusting for the systemic parameters of higher pulse rate, higher prevalence of arterial hypertension, higher blood concentration of triglycerides and cholesterol, higher frequency of drinking alcohol and body mass index. As a corollary, higher estimated CSFP was significantly associated with higher IOP after adjusting for the systemic parameters of rural region of habitation, higher systolic blood pressure, higher pulse rate, taller body height, higher blood concentration of cholesterol, and higher level of education.

As in previous studies, higher IOP was associated with higher blood pressure, higher pulse rate and/or higher prevalence of arterial hypertension [1]–[3], [5], [6], [15], [16]. In addition to these relationships, our study showed that higher IOP was also associated with higher estimated CSFP. These results fit with the hypothesis that the pressures in all three fluid-filled body compartments, i.e. the arterial blood system, the brain and the eye, are positively correlated with each other [17]. The results of our observational population-based study confirm a previous small-scaled interventional clinical study on patients who underwent direct lumbar CSFP measurement for neurological reasons and for whom the final neurological diagnosis made it unlikely that the neurological disorder had influenced the CSFP [1]–[3]. The results of our large-scaled present study thus compliment the previous investigation, with the advantage of a large and unselected study population and the disadvantage of not directly measuring the CSFP. The results are in agreement with the findings of a previous investigation on a study population in rural Central India [18].

The associations of IOP with the systemic parameters, in particular with estimated CSFP, were more interesting than the known relationships between IOP and ocular parameters. The relationship between IOP readings and central corneal thickness confirms multiple previous studies by clinicians and theoretical and practical considerations by Goldmann himself [7], [11], [12]. Interestingly, the anterior corneal refractive power additionally influenced the intraocular pressure readings: The higher the corneal refractive power was, i.e., the steeper the cornea was, the higher were the intraocular pressure readings. It agrees with the recent Central India Eye and Medical Study, and contradicts findings from the Reykjavik Eye Study, in which corneal curvature was not significantly associated with the intraocular pressure readings [9], [11]. The association between corneal curvature and IOP readings may be due to geometry, since a flat structure as compared with a steep structure needs less pressure to be flattened up to a standardized applanation area. The observation may have clinical implications, in particular for eyes after corneal refractive surgery which leads to flattening of the corneal surface. Refractive corneal surgery for correction of myopia may thus have two reasons for an underestimation of the IOP: Thinning of the cornea and flattening of its surface. The finding in our study that IOP was related with the shallowness of the anterior chamber is in agreement with previous population-based studies such as those by Foster and colleagues and others [10]. The correlation between IOP and axial myopia as found in our multivariate analysis agrees with previous investigations, but not with findings from the Los Angeles Latino Eye Study [12], [16], [19].

Our study was in agreement with results reported from other investigations on CSFP or an estimated CSFP. In the Central India Eye and Medical Study as in the Beijing Eye Study, higher estimated CSFP was associated with higher IOP [17]. These studies also suggested that estimated CSFP was associated with other ophthalmic parameters and conditions, such as ocular hypertension and open-angle glaucoma, subfoveal choroidal thickness, retinal vein diameter, and diabetic retinopathy [17], [18], [20]–[22].

The question arises which mechanism may be responsible for the potential association between CSFP and IOP. In a recent experimental investigation, Samuels and colleagues microinjected bicuculline methiodide which is a GABA-(gamma-amino-butyric-acid)-A receptor antagonist into the dorsomedial and perifornical hypothalamus in rats [23]. They found that the chemical stimulation of the hypothalamic region led to increases in heart rate, mean arterial blood pressure, IOP and CSFP. Interestingly, the peak in IOP increase occurred significantly later than the peak in CSFP leading to marked changes in the trans-lamina cribrosa pressure difference. Samuels and colleagues concluded that the neurons of the dorsomedial and perifornical hypothalamus might be a key effector pathway for the regulation of the autonomic tone by the suprachiasmatic nucleus and may be involved in a central regulation of CSFP, blood pressure and IOP. Other or additional possibilities could be to consider the arterial blood pressure as the driving force which may influence the production rate of cerebrospinal fluid pressure and the production rate of aqueous humour in a parallel manner. Additionally, the episcleral venous pressure may play a role since it may also depend on the CSFP.

Care must be taken not to transfer the result of our study performed on neurologically normal individuals onto neurologically abnormal patients, in whom a neurophysiologic reason is the likely cause of abnormal CSFP. In that case, the physiological relationships between CSFP, IOP and blood pressure may potentially no longer prevail. In a previous study by Saijadi and colleagues on 50 patients (pseudotumor cerebri: n = 14 patients; bacterial meningitis, n = 7; multiple sclerosis, n = 7), CSFP as measured by direct lumbar puncture was strongly correlated with IOP [24]. In contrast, Han and coworkers in a retrospective analysis of the clinical charts of 55 neuro-ophthalmological patients did not detect a significant correlation between IOP and CSFP [25]. Future studies appear necessary to further elucidate the associations between CSFP, IOP, blood pressure, body mass index and age in patients with neurological disorders as well as in normal individuals.

Potential limitations of our study should be mentioned. First, the whole statistical analysis depended on the formula to calculate the CSFP. The pilot study, in which the basis parameter for that formula were assessed included a relatively small number of subjects, and these subjects had a clinical reason to undergo lumbar puncture [3]. Although the neurological examination and the further clinical course revealed that it was unlikely that the lumbar CSFP measurement was markedly influenced by the reason to perform the lumbar puncture, one has to keep in mind, that the participants were not randomly selected normal subjects. The result of this formula was then termed CSFP and correlated with IOP as well as with other factors. Although the estimated CSFP was primarily just the result of a mathematical equation, the calculated CSFP values correlated well with invasively measured CSFP values in the independent test group in the pilot study. Nonetheless, the unknown general validity of the equation to estimate the CSFP may be the most important limiting factor of our study. Second, as for any population-based study, the rate of non-participation or non-availability of examination results can matter. In our study, the participation rate was 78.8% what may be acceptable. Third, IOP was measured only once, so that the question arises how representative this single IOP measurement was for the subjects IOP in general. Although the single IOP measurements may have increased the noise (or decreased representativeness) of the measurements, the associations in the multivariate analysis were statistically significant, so that this limitation in the study design may serve to strengthen the conclusions of the study. Fourth, it must be considered that the coefficients for the relationships between intraocular pressure and some other parameters were relatively low. Although these relationships were statistically significant with a *P*-value of <0.05, the low coefficients showed that only a fraction of the variability of intraocular pressure could be explained by that relationship. Future studies may address dynamic aspects of the association between IOP and CSFP based on the previous study by Morgan and coworkers [26], [27].

In conclusion, IOP is positively associated with estimated CSFP after adjusting for other systemic and ocular parameters. As a corollary, higher estimated CSFP is significantly associated with higher IOP in multivariate analysis. It agrees with previous studies with direct lumbar CSFP measurements. It fits with the hypothesis that the pressures in all three fluid body compartments (arterial blood system, brain compartment, intraocular space) are physiologically correlated with each other.
